# Artificial sphincter “BR - SL - AS 904” in the treatment of urinary incontinence after radical prostatectomy: efficacy, practicality and safety in a prospective and multicenter study

**DOI:** 10.1590/S1677-5538.IBJU.2018.0128

**Published:** 2018

**Authors:** Salvador Vilar Correia Lima, Evandilson Guenes Campos de Barros, Fabio de Oliveira Vilar, Flavia Cristina Morone Pinto, Thomé Décio Pinheiro Barros, José Carlos Truzzi, Luiz Gustavo M. de Toledo, Francisco Kanasiro, João Luiz Amaro

**Affiliations:** 1 Serviço de Urologia, Hospital das Clínicas, Universidade Federal de Pernambuco, UFPE, Recife, PE, Brasil;; 2 Departamento de Cirurgia, Centro de Ciências da Saúde, Universidade Federal de Pernambuco, UFPE, Recife, PE, Brasil; 3 Departamento de Urologia, Instituto do Câncer Dr. Arnaldo Vieira de Carvalho, São Paulo, SP, Brasil;; 4 Santa Casa de Misericórdia de São Paulo, São Paulo, SP, Brasil; 5 Serviço de Urologia, Hospital Santa Marcelina, Porto Velho, RO, Brasil;; 6 Disciplina de Urologia, Unesp - Universidade Estadual Paulista, São Paulo, SP, Brasil

**Keywords:** Urinary Incontinence, Urinary Sphincter, Artificial, Prostatic Neoplasms

## Abstract

**Purpose:**

The objective of the present study is to test the efficiency and practicality of a new artificial sphincter “BR - SL - AS – 904” in the control of urinary incontinence in post - PR patients and to evaluate their complications.

**Patients and Methods:**

Fifteen patients with incontinence after one year of radical prostatectomy were included prospectively. All patients underwent artificial urethral sphincter (AUS) implant “BR - SL - AS – 904” according to established technique. Independent variables such as free urinary flow, PAD weight test, ICIQ - SF score and urinary symptoms through the IPSS score were compared in different follow-up moments.

**Results:**

Patients submitted to AUS implantation did not present trans - operative or post - operative complications related to the surgical act such as: infection, hematoma, erosion or urinary retention. Device was inert to the body during the follow-up, showing an excellent adaptation of the patients, besides the easy handling. The mean age was 68.20 years 40% of the patients had systemic arterial hypertension, 6.7% diabetes mellitus, 6.7% were hypertensive and diabetic, 13.4% were hypertensive, had diabetes and hypercholesterolemia and 26.7% patients had no comorbidities. It was evidenced that the urinary flow peak during the follow-up remained stable. Decreased averages and median PAD weight test were 135.19 to 75.72 and 106.00 to 23.50, respectively. The IPSS score decreased and the quality of life increased (12.33 to 3.40 and 2.50 to 3.20 respectively). The ICQF - SF questionnaire score also showed a decrease, ranging from 16, 71 to 7.33.

**Conclusion:**

The artificial sphincter implant “BR - SL - AS 904” was reproducible, safe and effective in the control of urinary incontinence in post - PR patients.

## INTRODUCTION

Prostate cancer is the most frequently diagnosed non - skin cancer in the United States and the third leading cause of cancer deaths. In 2017, 1.688.780 new cancer cases and 600.920 cancer deaths were projected to occur in the United States (1). Radical prostatectomy (PR) is the currently treatment with satisfactory cancer results in patients with localized or locally advanced prostate neoplasia without major comorbidities. However, the main complication PR - associated is the involuntary loss of urine, ie, urinary incontinence (UI). After one year of persistent UI pos - PR, the artificial sphincter implant is the main treatment option.

AMS 800 artificial sphincter is currently considered the gold standard treatment for men with UI. However, this system has high complexity for the implantation of the device. It is necessary to perform a high number of procedures to obtain satisfactory rates of urinary continence associated with acceptable rates of surgical complications (2). In a retrospective study, from January 1972 to September 2015, 27.096 cases were included from the AMS 800 implants, of which 5.723 required either revision or explantation (21.1%). Younger age and penoscrotal approach were associated with higher device explantation and revision rates, while use of a tandem cuff was associated with higher explantation rates (3). In addition, another limiting factor for this type of device is that the operating mechanism is static, ie, the pressure exerted on the urethra through the cuff is constant as the patient is at rest, exercising, coughing or performing maneuvers that increase abdominal pressure (personal findings). Finally, those implants have a high economic cost making its accessibility quite limited, especially for those patients assisted in the public health system.

The BR - SL - AS 904 sphincter was developed in order to reduce the restrictions of the currently available devices, to improve the postoperative results and to make the implantation of an artificial urinary sphincter feasible and accessible.

## PATIENTS AND METHODS

This was a prospective, multicenter, non-randomized trial with patients with urinary incontinence after radical retropubic prostatectomy and submitted to artificial urethral sphincter implant “BR - SL - AS – 904”. The trial was carried out in accordance to the National Council of Health, the Helsinque Declaration and the Nuremberg Code for human experiment. The study is also listed on www.clinicaltrials.gov, and was approved by the National Ethics Committee in Research (CONEP # 814.933). The non - inclusion of a control group was discussed and accepted by the Ethical Committee of the Institution.

### Patient eligibility criteria

Fifteen patients with moderate and severe incontinence after one year of radical prostatectomy were included prospectively. All patients underwent artificial urethral sphincter (UE) implant “BR - SL - AS – 904” according to established technique. Patients submitted to previous radiotherapy; patients with urethral stenosis or previously submitted to internal urethrotomy due to vesico - urethral anastomosis stenosis were excluded.

### BR - SL - AS 904 sphincter appearances

**Figure 1:**
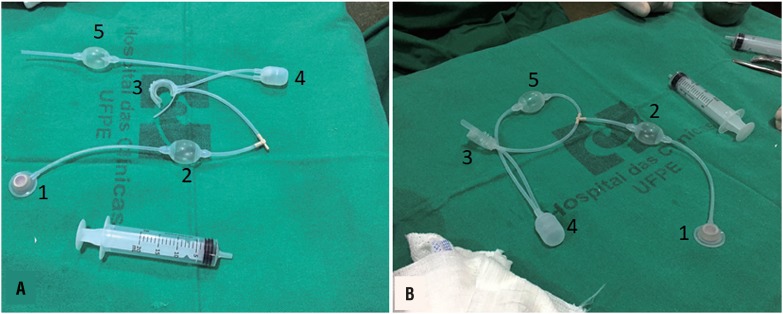
Appearance of the disassembled BR-SL-AS 904 sphincter (A), consisting of the following parts: 1; 2; 4; and 5: constriction-pumping system and 3: activating valve. B) sphincter ready to be implanted after air removal by injecting 15ml of saline through the activator valve (B).

**Figure 2:**
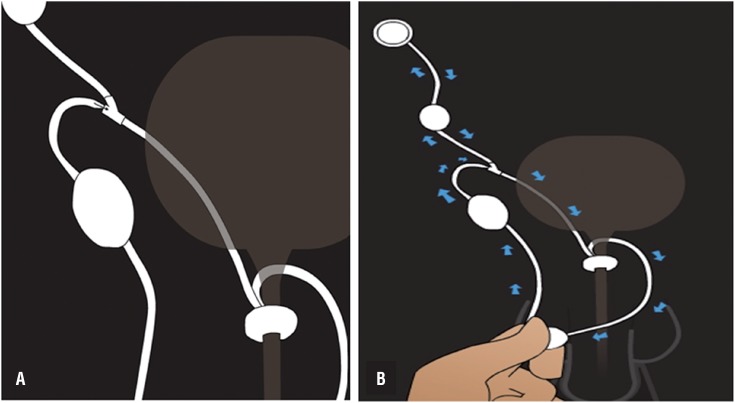
A) Pressure reducing system and B) Operating system. The resting device maintains urethral compression preserving urinary continence. (A). During pumping, the fluid present in the apparatus is displaced from the urethral cuff to the reservoir located in the peritoneal cavity and through a flow reducing system it slowly returns to the cuff, causing it to remain deflated for about three minutes allowing urination (B).

The proposed device operating mechanism including two parts: constriction - pumping system and activating valve (Figure-1). The resting device maintains urethral compression preserving urinary continence. During pumping, the fluid present in the device is displaced from the urethral cuff to the reservoir located in the peritoneal cavity and through a flow reducing system it slowly returns to the cuff, causing it to remain deflated for about three minutes allowing urination (Figure-2).

### Surgical technique and device's implant

**Figure 3:**
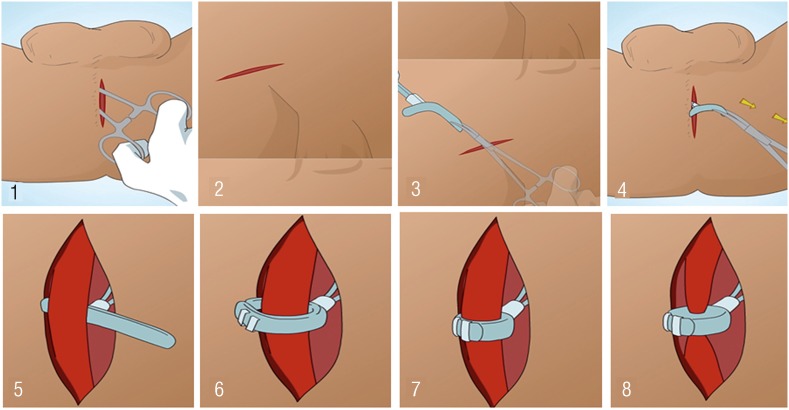
Surgical technique and device implantation steps: 1-2) Perineal and inguinal incision, respectively; 3) Inguinal tunnel construction and device passage; 4) Device in the perineal region; 5-8) Periurethral cuff being activated and locked.

**Figure 4:**
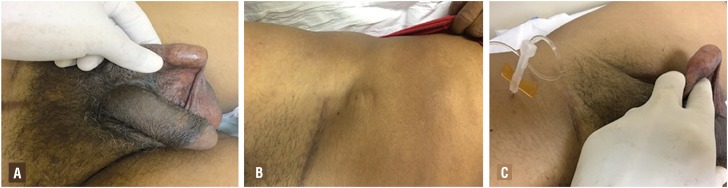
Aspect of the sphincter BR-SL-AS 904 (Pump), activation valve (B). Surgical stitches were removed on the tenth postoperative day and the device activated on the thirtieth day by infusing 15 ml of distilled water into the system by the activation valve (C).

The implant of the device was performed through two incisions: one perineal and one inguinal. In the perineal incision, a five - centimeter incision was made at the bulbar urethra level, allowing the passage of the constrictor balloon. After the cuff is passed, it is locked through the safety catch. After the perineal surgical time, an inguinal incision was made close to the external inguinal ring, dissected by anatomic planes, with communication between the inguinal and perineal incisions, parallel to the inguinal canal, forming a tunnel through which the device is carefully introduced. In the inguinal region, an incision was made in the aponeurosis of the external oblique muscle to gain access to the Retzius space. This space is dissected carefully forming a retropubic virtual cavity, where the reservoir of the urethral sphincter would be placed. After the reservoir was allocated in the Retzius space, a new subcutaneous tunnel was made towards the patient's flank. After passage of the device through the conduit formed we carefully allocate the periurethral cuff around the bulbar urethra and lock the urethral cuff using the safety clips (Figure-3). The wound suture was performed by planes with vicryl 3 – 0 and skin with 4 – 0 mononylon. The surgical stitches were removed on the tenth postoperative day and the device activated on the thirtieth day by infusing 15 mL of distilled water into the system through the activation valve. After activation of the system, the patient was instructed to manually activate the pump located in the scrotal sac to start voiding (Figure-4).

### Clinical and urological outcomes

As an independent variable, the implantation of the device was evaluated, and as independent variables: free urinary flow, PAD weight test, quality of life through ICIQ SF score; urinary symptoms through the IPSS score, all of them in the different moments of the urologist evaluations and lastly possible complications inherent to the implantation of the device such as infections, erosions and device failure.

### Statistical analyses

Data were analyzed descriptively and inferentially. The descriptive analysis was through absolute frequencies in the categorical variables and the statistics: mean and standard deviation. The inferential analysis was performed through the F (ANOVA) tests for repeated measures and Friedman's test for the comparison between the evaluations. In the case of significant difference by the F test (ANOVA), multiple Bonferroni comparisons were obtained. The verification of the normality hypothesis was through the Shapiro - Wilk test. The margin of error used in the statistical test decisions was 5%. The data were tabulated in Excel^®^ and analyzed in the SPSS (Statistical Package for the Social Sciences, IBM^®^ statistics, version 23.0).

## RESULTS

Fifteen patients underwent implantation of AUS at the three centers participating in the study. The mean age was 68.2 ± 7.5 years. In comorbidities analysis, it was observed that 40% of the patients had hypertension, 6.7% presented diabetes mellitus, 6.7% were hypertensive and diabetic patients, 6.7% had chronic renal insufficiency, 13.4% were hypertensive, diabetic and had hypercholesterolemia, and 26.7% of the patients had no comorbidities. The mean postoperative follow-up was 192.71 months. These data are described in Table-1.

**Table 1:** Mean age and medical comorbidities.

Outcomes		Total
**n**		**15**
Age (years): mean ± SD		68.20 ± 7.56
**Comorbidity**		
	Chronic Renal Insufficiency	1
	Hypertension	6
	Hypertension + Diabetes Mellitus	1
	Hypertension + Diabetes Mellitus + Dyslipidemia	2
	Diabetes Mellitus	1
	None	4
**Satisfaction rate**		
	Unsatisfied	15
	Post-surgery (weeks)	192.71 ± 100.88

Analyzing the complaints of the patients studied, it was identified that two thirds of them did not have voiding urgency, this index remained stable until the end of the study. The percentage of patients classified with the light score on the IPSS scale was 40.0% at visit 1, 46.7% after device activation and 80.0% at visit 4. The percentage of those classified as moderate had a variation of 33.3% at visit 1 to 20% at visit 4. The severe classification in the IPSS was 26.7% in the pre - op, and zero at visit 4. In the analysis of the quality of life an improvement was identified in “well and happy”, as well as a decrease in “unhappy and terrible” scores during follow-up visits. Data described in Table-2.

**Table 2:** Urinary urgency, IPSS and quality of life per urologist visit.

		Urologist visit				
		Pre-op	Visit 1	Visit 2	Visit 3	Visit 4
Outcome		n	n	n	n	n
TOTAL		15	15	15	15	15
**Urinary urgency**						
	Yes	5	4	6	3	1
	No	10	11	9	10	9
	Not informed	–	–	–	2	5
**IPSS**						
	Mild (0 – 7)	6	8	7	9	12
	Moderate (8 – 19)	5	5	7	5	3
	Severe (20 – 35)	4	2	1	1	–
**Quality of life**						
	Unhapy	4	4	1	3	1
	Awful	4	3	1	1	2
	Discomfort	3	–	4	4	3
	Generally well	1	7	3	4	3
	Regular	2	1	3	1	–
	Happy	–	–	2	–	1
	Nor informed	1	–	1	2	5

The Outpatient Follow-up was performed every 30 days (Visit).

**Figure 5:**
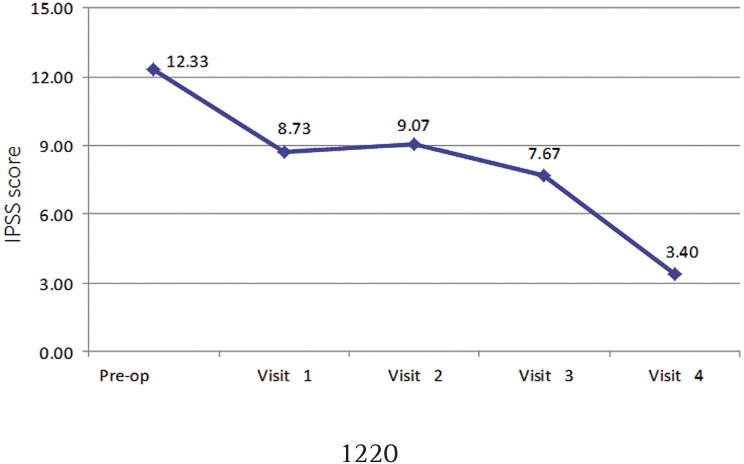
IPSS score per medical evaluation.

Table-3 presents the statistics related to the different clinical and urological variables. Patient's weight and BMI remained stable during the course of the study, as well as the number of daily urinary frequency and free peak flow. It was possible to verify a statistically significant difference (p = 0.016) in the IPSS score: highest mean in the preoperative period (12.3) and the lowest in the visit 4 (3.4) (Figure-5). Although there was no statistical relevance for the other variables, it was observed an objective improvement in the scores of involuntary urinary loss and improvement of quality of life with maintenance of urinary flow force without obstructive symptoms.

**Table 3:** Statistics related to the different clinical and urological variables.

	Urologist evaluation					
Outcome	Pre-op	Visit 1	Visit 2	Visit 3	Visit 4	P-value
Weight	74.56 ± 16.06	69.50 ± 10.80	78.20 ± 17.00	76.20 ± 14.14	74.87 ± 7.33	p(1) = 0.420
BMI	26.61 ± 4.09	25.19 ± 3.57	26.91 ± 4.24	26.96 ± 3.74	26.59 ± 3.38	p(1) = 0.487
Daily urination	5.47 ± 2.95	4.87 ± 2.50	4.07 ± 2.76	5.85 ± 1.68	4.89 ± 1.27	p(2) = 0.206
Urinary free flow	19.30 ± 7.44	21.89 ± 11.04	16.97 ± 7.90	16.27 ± 8.70	21.14 ± 11.35	p(1) = 0.812
PAD test	135.19 ± 159.54	94.90 ± 77.15	162.53 ± 217.53	110.37 ± 126.90	75.72 ± 95.29	p(2) = 0.092
IPSS score	12.33 ± 7.57^(A)^	8.73 ± 6.08^(AB)^	9.07 ± 7.20^(AB)^	7.67 ± 6.62^(B)^	3.40 ± 3.92^(C)^	*p(1) = 0.016
Quality of life	2.50 ± 1.40	2.87 ± 1.46	3.86 ± 1.46	2.92 ± 1.32	3.20 ± 1.40	p(2) = 0.266
ICIQ-SF	16.71 ± 2.69	14.20 ± 5.78	10.47 ± 7.1	11.47 ± 7.31	7.33 ± 7.17	p(1) = 0.126

* Mean P ≤ 0.05.

(1) One way ANOVA;

(2) Friedman's test. Different letter at the same line means statistically different.

The device was inert to the body throughout the initial follow-up of the study, showing an excellent adaptation of the patients. After clarification at the device activation visit, the patients were able to handle the device without difficulties, thus demonstrating its practicality. At the second follow-up visit, four patients reported a return of urinary loss and difficulty in activating the device, probably due to a mechanical problem. After information and clarification, two patients decided to exchange for a new device, while two other patients chose to remain with it. In the trans - operatory period, it was observed a fracture of the “T” connection hoses in both exchanged devices. Those complications occurred after the fourth month of surgery. There was no complication related to the surgical procedure or the reaction between the device and the organism, due to the mechanical problem (material fatigue). At the moment, these patients are in clinical follow-up according to established protocol. This event was communicated to the ethics committee of the participating center.

## DISCUSSION

Patients undergoing radical prostatectomy (RP) may suffer of urinary incontinence (UI) mainly due to damage sustained to the distal urethral sphincter, essentially producing stress urinary incontinence. In men, the artificial urinary sphincter (AUS) is currently considered the gold standard treatment for UI (4, 5). In our study, we proposed a new device for UI treatment after RP, through the analysis of the learning curve of the device implant, and through the dependent variables such as PAD weight test, ICIQ SF score, IPSS score and main related complications.

After more than 25 years of widespread use, the modern version of the AMS800 has proven to be a reliable surgical option for the management of non - neurogenic UI in men (6). However, large amounts of data regarding efficiency, complications, and patient satisfaction have been published after artificial sphincter implantation, but the quality of these reports does not meet current standards of evidence-based medicine (7-9). The AMS800 remains the gold standard but does not have the ability of rearrange the cuff in case of postoperative urethral atrophy (10, 11) and no option to adjust the issued pressure of the device after activation. The artificial sphincter BR - SL - AS 904, unlike the commercially available sphincters, has a pressure transmission system, where a reservoir in the patient's abdominal cavity has a direct connection with the urethral cuff. Therefore, the pressure exerted on the reservoir is transmitted through the hydraulic system to the urethral cuff, maintaining the continence of the patient only during stress. This device had already been described experimentally and the authors suggests that the direct pressure transmission to the cuff is an interesting concept to improve clinical outcomes of hydraulic sphincters (6). All surgeons reported the implantation of BR - SL - AR 908 being straightforward, fast, feasible and reproducible, with also a short learning curve to achieve mastery (personal findings). Unlike the facility offered by our device, the gold standard sphincter (AM800) is still provided in several boxes and separated components to assemble during the procedure with a constraining preparation (12). In addition, none of patients submitted to our implant presented trans - operative or postoperative complications related to the surgical procedure, using Clavien - Dindo score (13).

Mean age of the population at surgery was 68.2 years (SD 7.5) and did not appear to be related to any complications, however it is unclear why younger age would lead to higher complication rates. One potential explanation is that younger patients in this series had longer documented follow-ups, and consequentially a higher chance for complications and need for revision surgery. There are limited reports that have specifically examined the effect of age on AUS outcomes (14, 15). While one study did demonstrate that octogenarians were more likely to experience erosion or infection compared with younger patients (28), the 5 - year device survival rates were comparable to those reported in younger men (63% to 70%). As such, current evidence remains unclear to recommend making decisions on AUS placement and outcomes solely based on age.

We sought to identify risk factors for AUS complications such as prior radiation, or comorbid conditions as diabetes and hypertension. This study could not identify any statistically significant risk factors for AUS complications. To do so, it will be necessary a higher number of patients and studies. However, we could notice in our study that patient comorbidities, in particular diabetes (40%) and hypertension + diabetes (13%), had similar rates of urinary urgency, IPSS and reported quality of life. In particular, pelvic radiation and high blood pressure have been considered potential risk factors for AUS treatment failure and complication but our study did not have any case of it (16, 17).

In general, there was an improvement in urinary urgency (5 patients at pre - op and only 1 at fourth visit); better IPSS score (6 patients with mild score at pre - op and more 6 at visit 4) and finally a better quality of life (just one patient reported being unhappy at visit 4). Assessment of urinary continence was based on daily urination, urinary free flow, PAD weight test, IPSS score, quality of life and ICIQ - SF values. Although not statistically significant when the comparative analysis tests were applied over the following-up, an improvement in the scores of involuntary urinary loss and quality of life was observed with preservation of urinary flow force without the occurrence of obstructive symptoms. Previous studies assessed functional and quality of life outcomes by PAD use or no validated questionnaires measuring urinary incontinence, frequency and nocturia but did not address long - term functional outcomes for health related quality of life (18-20). Our results are similar to other series with patients reporting an improvement in urinary incontinence and quality of life.

In our study, four different devices (26.7%) had mechanical problem and needed to be removed. However, from 15 cases those four were the only where complication were found. Recently a retrospective study identified 27.096 cases of which the main complications were: erosion of the cuff (4%); loss of fluid (3.8%); cuff atrophy (2.4%); infections (8%) and herniation of the pump (0.2%) (3). In contrast, there were no cases of infection related to device implantation during the follow-up period of this study. There was also no evidence of urethral erosion or appliance extrusion. The device was inert, without triggering inflammatory reactions or infectious processes. Another study analyzing 1.082 artificial sphincter implants followed up for 4.2 years, re - operated 125 patients (11.6%) due to malfunction of the device (21). The urethral cuff was the component that failed the most (46.1%), followed by the abdominal reservoir (22.6%) and the pump (9.6%). Rupture of the tubes were not observed. As the rupture of the ducts is an uncommon complication and the number of patients analyzed was low, we believe that after an improvement in the quality of the T - connection, it would be possible to promote satisfactory urinary continence. However, larger cohort studies with longer follow-up are needed to assess the device efficacy and safety.

## CONCLUSIONS

According to the present study, the artificial sphincter implant BR - SL - AS 904 was reproducible, safe and efficient in the control of urinary incontinence in patients after radical prostatectomy. Improvements in both quality and of the implant material and increase the number of patients can make this treatment modality very attractive and widely practiced.
